# Fine-Mapping of Immunodominant Linear B-Cell Epitopes of the Staphylococcus Aureus SEB Antigen Using Short Overlapping Peptides

**DOI:** 10.1371/journal.pone.0090445

**Published:** 2014-03-05

**Authors:** Zhuo Zhao, Bin Li, He-Qiang Sun, Jin-Yong Zhang, Yi-Lin Wang, Li Chen, Jian Hu, Ya-Fei He, Hao Zeng, Quan-Ming Zou, Chao Wu

**Affiliations:** 1 National Engineering Research Center of Immunological Products, Department of Microbiology and Biochemical Pharmacy, College of Pharmacy, Third Military Medical University, Chongqing, PR China; 2 Department of Gastroenterology, Xinqiao Hospital, Third Military Medical University, Chongqing, PR China; Rockefeller University, United States of America

## Abstract

Staphylococcal enterotoxin B (SEB) is one of the most potent Staphylococcus aureus exotoxins (SEs). Due to its conserved sequence and stable structure, SEB might be a good candidate antigen for MRSA vaccines. Although cellular immune responses to SEB are well-characterized, much less is known regarding SEB-specific humoral immune responses, particularly regarding detailed epitope mapping. In this study, we utilized a recombinant nontoxic mutant of SEB (rSEB) and an AlPO_4_ adjuvant to immunize BALB/c mice and confirmed that rSEB can induce a high antibody level and effective immune protection against MRSA infection. Next, the antisera of immunized mice were collected, and linear B cell epitopes within SEB were finely mapped using a series of overlapping synthetic peptides. Three immunodominant B cell epitopes of SEB were screened by ELISA, including a novel epitope, SEB_205-222_, and two known epitopes, SEB_97–114_ and SEB_247-261_. Using truncated peptides, an ELISA was performed with peptide-KLH antisera, and the core sequence of the three immunodominant B cell epitopes were verified as SEB_97-112_, SEB_207-222_, and SEB_247-257_. In vitro, all of the immunodominant epitope-specific antisera (anti-SEB_97-112_, anti-SEB_207-222_ and anti-SEB_247-257_) were observed to inhibit SEB-induced T cell mitogenesis and cytokine production from splenic lymphocytes of BALB/c mice. The homology analysis indicated that SEB_97–112_ and SEB_207-222_ were well-conserved among different Staphylococcus aureus strains. The 3D crystal structure of SEB indicated that SEB_97–112_ was in the loop region inside SEB, whereas SEB_207-222_ and SEB_247-257_ were in the β-slice region outside SEB. In summary, the fine-mapping of linear B-cell epitopes of the SEB antigen in this study will be useful to understand anti-SEB immunity against MRSA infection further and will be helpful to optimize MRSA vaccine designs that are based on the SEB antigen.

## Introduction

Methicillin-resistant Staphylococcus aureus (MRSA) infections cause a high mortality rate in the United States [1], China [2] and Japan [3]. MRSA infections have been the leading cause of death by a single infectious agent in the USA, exceeding deaths that are caused by HIV/AIDS [4]. However, no effective MRSA vaccine has been successfully developed thus far [5]. Approximately 50% to 80% of isolated Staphylococcus aureus (S. *aureus*) strains show enterotoxin activity [6]–[7]. Staphylococcal enterotoxin B (SEB) is one of the most common and potent exotoxins of S. *aureus* [8] and is considered to be a primary causative agent of staphylococcal TSS(Toxic Shock Syndrome) [9]–[10].

The pathophysiological role of SEB in human diseases, such as atopic dermatitis [11] and asthma [12], has recently been recognized. Unfortunately, prophylactic or therapeutic treatments for this potential threat have yet to be developed. Many researchers have generated monoclonal antibodies against SEB that could prevent toxic shock syndrome that is induced by S. *aureus* infections [13]–[17]. Although SEB has a conserved sequence and stable structure, SEB is a potent toxin that acts as a lethal and incapacitating agent [18]–[19], which has limited native SEB as a vaccine candidate. In our study, we found that a nontoxic mutant of SEB (rSEB) could induce effective immune protection against MRSA in a BALB/c mouse model.

Previous studies demonstrated that antibody responses play a major protective role in specific immunity against MRSA [20]. A recent study regarding SEB provided further evidence for the important role of SEB in S. *aureus* infection and a rationale for anti-SEB IgG as an immunotherapeutic agent for severe staphylococcal diseases [21]. However, without knowing the particular detailed epitope-mapping of SEB, the specific molecular basis and mechanism of the humoral immune protective response to SEB in MRSA infection remain unclear, unlike the cell immune responses to SEB, which have recently been studied in detail [22]–[23].

To gain insight regarding the anti-SEB humoral response and to further characterize detailed antibody epitopes, we used synthetic overlapping peptides to investigate the SEB-specific antibodies in MRSA-infected mice that were administered rSEB vaccinations. The linear B-cell epitopes were mapped, and some minimal immunodominant epitopes of SEB were identified. Then, we evaluated the neutralization of SEB-induced responses by the immunodominant-epitope specific antisera. Furthermore, we confirmed the conservation of all three immunodominant epitopes, and we located these epitopes in a 3D structural model of SEB. Our findings characterized three linear B-cell epitopes on the SEB of MRSA, which will be helpful for understanding anti-SEB immunity against MRSA infections and for developing MRSA vaccines.

## Results

### rSEB vaccination can protect BALB/c mice from MRSA252 infection

**Figure 1:**
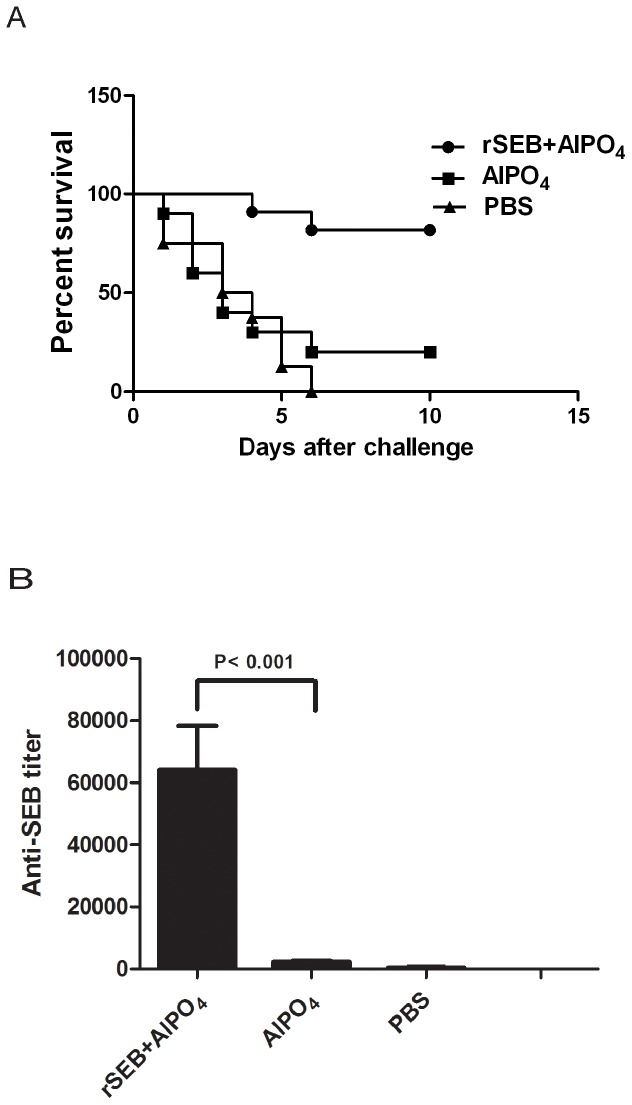
Percent survival and ELISA detection of antibody levels in immunized mice. (A) Female BALB/c mice were immunized with rSEB plus AlPO_4_ adjuvant, AlPO_4_ adjuvant alone or PBS alone before MRSA252 infection. Two weeks after the last immunization, mice were challenged with 8.4×10^8^ CFU of MRSA252 by intravenous injection. The percent survival was analyzed in mice after the MRSA challenge. (B) To determine the anti-SEB titers in immunized animal serum samples, microtiter plates were coated with native SEB. Sera from immunized mice were analyzed using an ELISA. The antibody levels in immunized mice with rSEB plus AlPO_4_ adjuvant, AlPO_4_ adjuvant alone or PBS alone are shown.

To determine the protective role of rSEB against MRSA infection, BALB/c mice were immunized using rSEB plus AlPO_4_ adjuvant, AlPO_4_ adjuvant alone or PBS alone before MRSA252 infection. In total, 80% of mice that were immunized with rSEB plus AlPO_4_ adjuvant survived without clinical signs, and the survival rate of these mice was higher than that in the AlPO_4_ adjuvant alone group (20%) and higher than that in the PBS group (none survived) (Figure 1A). To assess the SEB-specific antibody titer, one week after a booster immunization, the sera of immunized mice were examined using an ELISA. The results showed that the vaccination of rSEB plus AlPO_4_ adjuvant induced a significant antibody response that was specific to the native SEB in mice. The mean antibody titer in the rSEB plus AlPO_4_ adjuvant vaccine-immunized group was up to 1∶64000 higher than that in the AlPO_4_ adjuvant or than that in the PBS alone group (Figure 1B).

### Identification of the three immunodominant antigenic determinants on SEB by overlapping 18-mer peptide ELISA

**Figure 2:**
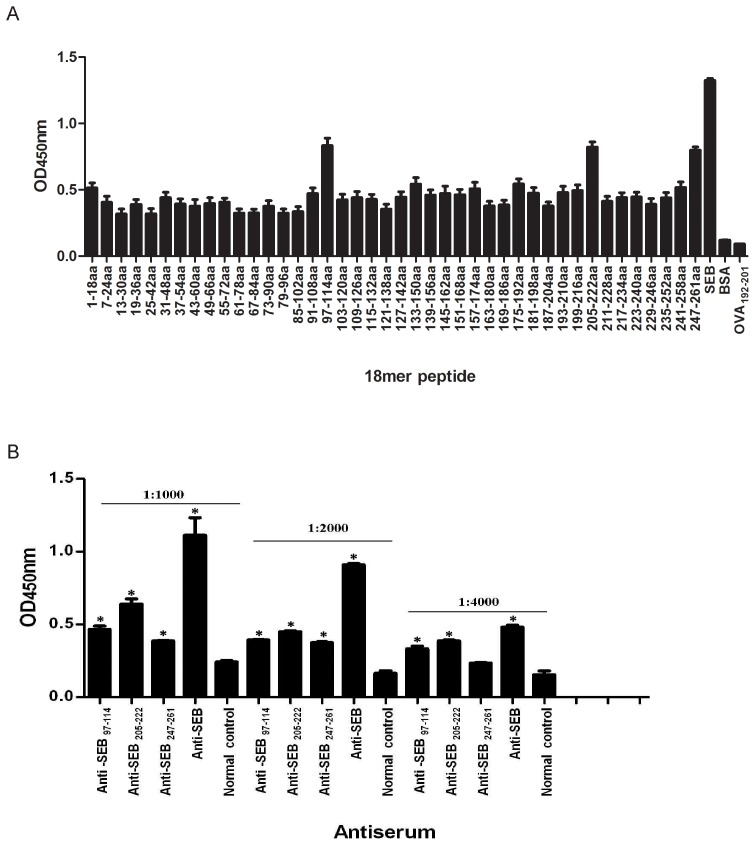
ELISA detection of B cell epitope mapping of SEB and anti-immunodominant peptide sera reactions with SEB. (A) B cell epitope mapping of SEB using an overlapping 18-mer peptide ELISA. To determine the immunodominant peptides of SEB, microtiter plates were coated with synthetic overlapping peptides that spanned the entire length of the SEB of MRSA252 or BSA and OVA_192-201_ (negative control peptides). Then, antiserum samples from BALB/c mice that were immunized with rSEB plus AlPO_4_ adjuvant were detected. The absorbance was read at 450 nm. The absorbances at 450 nm for the peptides SEB_205-222_, SEB_97–114_ and SEB_247-261_ were significantly higher than BSA (P<0.01) and higher than OVA_192-201_ (P<0.01). (B) Immunodominant peptide antiserum reaction with SEB using an ELISA. To confirm the reactivity of immunodominant peptide-specific antisera with SEB, BALB/c mice were immunized with KLH-conjugated immunodominant peptides and acquired anti-SEB_205-222_, SEB_97–114_ and SEB_247-261_, respectively. Microtiter plates were coated with native SEB. Then, all of the immunodominant peptide-specific antisera at the dilutions of 1:1000, 1:2000 and 1:4000 were detected using an ELISA. To statistically analyze the data in Figure 2B, we used an unpaired Student's t-test in the software SPSS 13.0. Each bar represents the means ± s.d. of triplicate measurements, and the data shown are representative of two or more independent experiments. At different dilutions, all of the peptide-specific antisera were stronger to bind SEB than normal sera of BALB/c mice (* P<0.05), particularly at the dilutions of 1:1000 and 1:2000 (P<0.01). When the dilutions of the peptide-specific antisera were expanded to 1:4000, the differences were not obvious.

To determine the immunodominant peptides of SEB, antiserum samples were collected from BALB/c mice that were immunized with rSEB plus AlPO_4_ adjuvant. Forty-two 18-mer overlapping peptides that spanned the entire length of the SEB of MRSA252 were synthesized. Linear B-cell epitope mapping of SEB was determined using an ELISA with overlapping 18-mer peptides. The results indicated that the strongest IgG antibody reactivity was concentrated on three major immunodominant peptides: SEB_97-114_ (NKNIDLFGTNYYYQCYFS), SEB_205-222_ (YETGYIKFIEGNGHSFWY) and SEB_247-261_ (VESKSINVEVHLTKK) of the SEB antigen (Figure 2A). Because the regions SEB_97-114_ and SEB_247-261_ of SEB have been previously reported to contain the B cell epitopes SEB_252-261_ (INVEVHLTKK) and SEB_96-103_ (KNKNIDLF) [24]–[27], respectively, SEB_205-222_ might contain a novel linear B-cell epitope of SEB.

To confirm the reactivity of anti-immunodominant peptide sera with SEB, antiserum samples from immunized BALB/c mice with KLH-conjugated immunodominant peptides were collected. Antibodies against three immunodominant peptides showed stronger reactivity with SEB than with normal sera at different dilutions (* P<0.05) (Figure 2B), particularly at the 1∶1000 and 1∶2000 dilutions (P<0.01). However, when the dilutions of the peptide specific antisera were expanded to 1∶4000, the difference was not so obvious. We thought the suitable dilution of the peptide-specific antisera was necessary for binding with SEB. Thus, all results indicated that peptides SEB_97-114_, SEB_205-222_ and SEB_247-261_ contained linear B-cell epitopes.

### Fine-mapping analysis of the immunodominant epitopes by its truncated peptides

**Figure 3:**
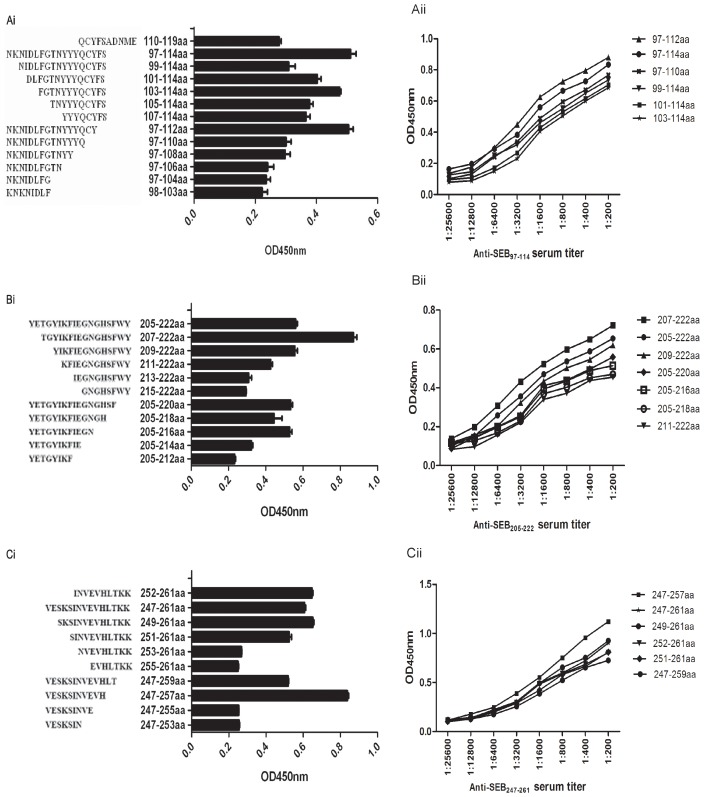
Fine-mapping analysis of the immunodominant peptides by their truncated peptides using an ELISA. To verify the core sequence within peptides SEB_97–114_, SEB_205-222_ and SEB_247-261_, we utilized the truncated peptide ELISA and the immunodominant peptide antisera titration analysis. The dilutions of the immunodominant peptide antisera were 1:200, 1:400, 1:800, 1:1600, 1:3200, 1:6400, 1:12800 and 1:256000. In Ai, Bi, and Ci, truncated peptides reacted with immunodominant peptide antisera. The peptides, (including immunodominant peptides, reported epitopes and truncated peptides, all of which were at the concentration of 5 µM) as indicated in each figure panel, were coated on ELISA wells. After washing and 5% skim milk blocking, 1:500 diluted immunodominant peptide antiserum was added to the wells for 1.5 h at 37°C, which was followed by standard ELISA analysis. In Aii, Bii and Cii, the core sequences were confirmed by a series dilution ELISA.

To finely map the immunodominant epitopes of SEB_97–114_, SEB_205-222_ and SEB _247-261_, peptides were gradually truncated from either the carboxy or the amino terminal for each immunodominant peptide. All the truncated peptides were scanned using an ELISA with 1∶500 diluted antisera from 18-mer peptide-immunized mice. The data are representative of at least 2 independent experiments, with error bars indicating the standard deviation. The results showed that, among the truncated peptides, SEB_97–112_, SEB_97–114_, SEB_97–110_, SEB_99–114_, SEB_101–114_ and SEB_97–112_ had stronger responses to the SEB_97–114_ antiserum (Figure 3Ai); SEB_205–222_, SEB_207–222_, SEB_209–222_, SEB_211–222_, SEB_205–220_, SEB_205–218_ and SEB_205–216_ had stronger responses to the SEB_205–222_ antiserum (Figure 3Bi); and SEB_247–261_, SEB_249–261_, SEB_252–261_, SEB_251–261_, SEB_247–259_ and SEB_247–257_ had stronger responses to the SEB_247–261_ antiserum (Figure 3Ci). To further confirm the core sequences of the three immunodominant epitopes, SEB_97–114_, SEB_205-222_ and SEB_247-261_, peptide antisera were diluted at different titers, and the truncated peptides with stronger responses to corresponding peptide antisera were once again scanned using an ELISA. The results showed that the strongest response to SEB_97–114_ corresponded with SEB_97-112_ (Figure 3Aii); SEB_207-222_ corresponded with SEB_205-222_ (Figure 3Bii); and SEB_247-257_ corresponded with SEB_247-261_ (Figure 3Cii).

### Effect of the immunodominant epitope antisera on reducing SEB-induced T-cell proliferation and cytokine production *in vitro*

**Figure 4:**
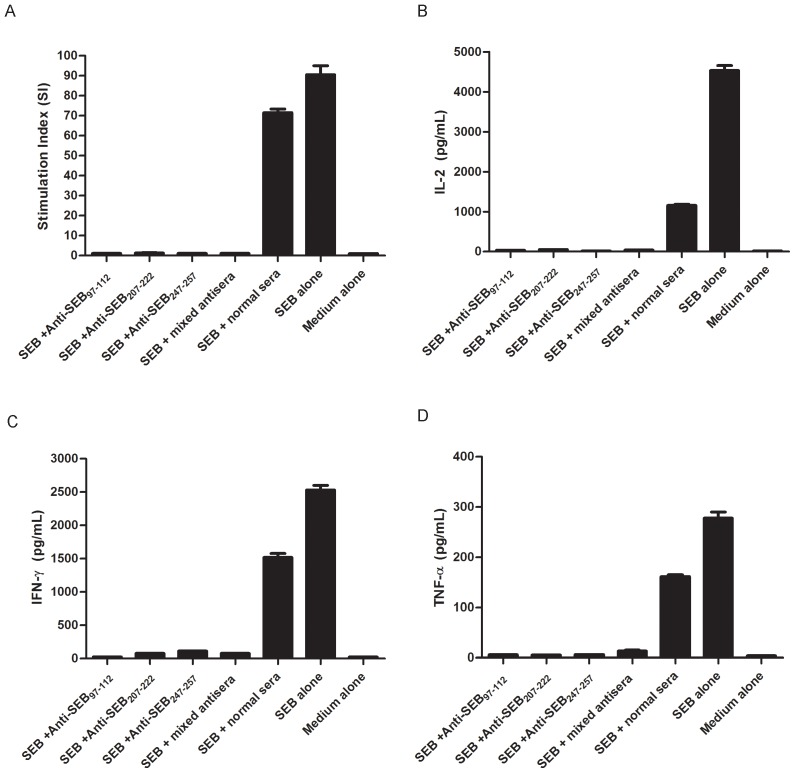
Neutralization/inhibition of SEB-mediated T-cell activation and cytokine production. BALB/c splenocytes were cultured as outlined in the Materials and methods section, with the following additives: Medium alone; SEB alone, 50 ng/ml SEB; SEB + Anti-SEB_97-114_, 50 ng/ml SEB+100 µg/ml Anti-SEB_97-112_ sera IgG; SEB + Anti-SEB_207-222_, 50 ng/ml SEB+100 µg/ml Anti-SEB_207-222_ sera IgG; SEB + Anti-SEB_247-257_, 50 ng/ml SEB+100 µg/ml Anti-SEB_247-257_ sera IgG; SEB + mixed antisera, 50 ng/ml SEB+33.3 µg/ml Anti-SEB_97-112_ sera IgG +33.3 µg/ml Anti-SEB_207-222_ sera IgG +33.3 µg/ml Anti-SEB_247-257_ sera IgG; SEB +normal sera, 50 ng/ml SEB+100 µg/ml normal sera IgG. Each bar represents the means ± s.d. of triplicate measurements, and the data shown are representative of two or more independent experiments. All of the immunodominant epitope-specific antisera could significantly inhibit SEB-induced T cell mitogenesis (Figure 4A) and significantly inhibit SEB-induced IL-2 (Figure 4B), IFN-γ (Figure 4C) and TNF-α (Figure 4D) production from splenocytes of BALB/c mice (P<0.01).

The ability of the immunodominant peptide antisera to inhibit SEB-induced T-cell proliferation was examined in vitro in BALB/c mouse splenocytes. The results of the experiments revealed that all of the immunodominant peptide antisera significantly (P<0.01) neutralized SEB-induced T-cell proliferation and IL-2, IFN-γ and TNF-α production from splenocytes compared with cells treated with SEB and normal sera or SEB alone (Figure 4A–D).

### Sequence alignment and localization of immunodominant epitopes on SEB

**Figure 5:**
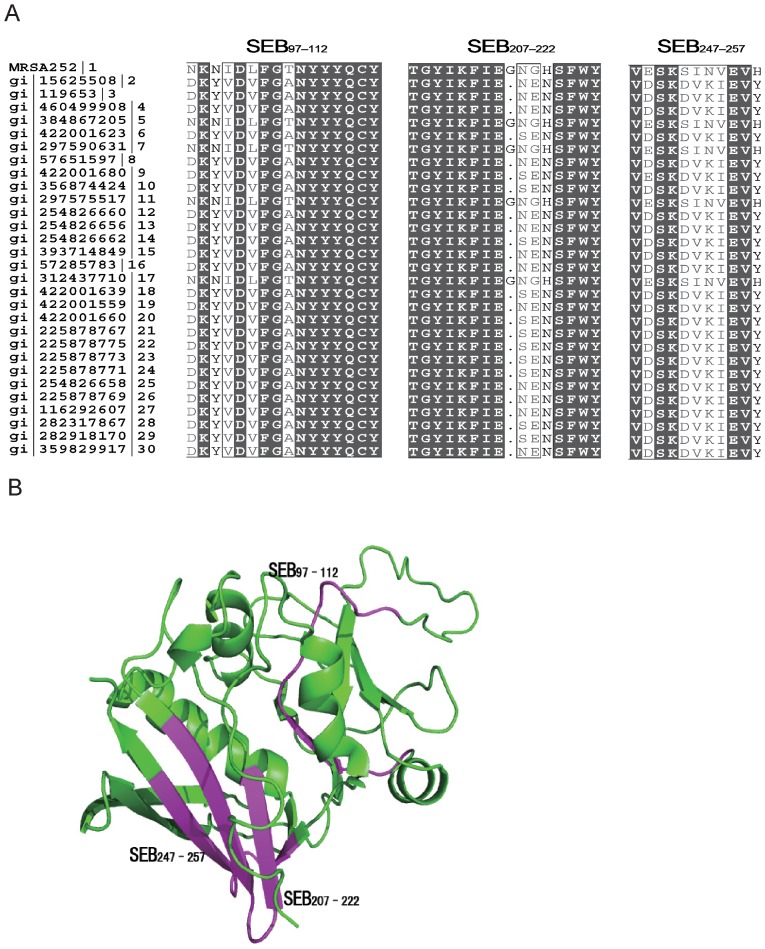
Sequence alignment and localization of immunodominant epitopes on SEB. (A) Sequence alignment of immunodominant peptides of SEB demonstrates that the amino acids of SEB_97–112_, SEB_207-222_ of SEB were conserved in all S. *aureus* strains, whereas SEB_247-257_ was not. (B) Localization of immunodominant peptides SEB_97–112_, SEB_207-222_, and SEB_247-257_ on the 3D crystal structure of SEB. SEB_97–112_, SEB_207-222_, and SEB_247-257_ are shown in red, and the other sequences of SEB are shown in green.

To determine the conservation of immunodominant epitopes, the amino acid sequences of SEB_97–112_, SEB_207-222_ and SEB_247-257_ of SEB from the selected MRSA252 strain and another 29 S. *aureus* strains were retrieved from the GenBank database for alignment. The results revealed that sequences of two immunodominant epitopes were highly conserved among S. *aureus* strains, with the amino acid identities of SEB_97–112_ and SEB_207-222_ over 70%, whereas the amino acid identity was less than 50% for SEB_247-257_ (Figure 5A). Meanwhile, three immunodominant epitopes were mapped in the 3D crystal structure of SEB (PubMed protein database) using the PyMOL 1.1 program. These epitopes are shown in red, indicating SEB_97–112_, SEB_207-222_ and SEB_247-257_, and the other SEB sequences are shown in green (Figure 5B). The result showed that SEB_97–112_ was in the loop region inside SEB, whereas SEB_207-222_ and SEB_247-257_ were in the β-slice region outside SEB.

## Discussion

In this study, we confirmed rSEB as a MRSA vaccine candidate for humans. Then, we finely mapped and characterized immunodominant linear B cell epitopes on the SEB of MRSA using overlapping 18-mer peptides, which included a novel epitope, SEB_205-222_, and two known epitopes, SEB_97–114_ and SEB_247-261_. The most potent core sequences were determined using truncated peptides that were based on the dominant 18-mer peptide. Then, the neutralization of SEB-induced responses by the immunodominant-epitope specific antisera was detected in vitro. The homology analysis indicated that SEB_97–112_ and SEB_207-222_ were well-conserved among different Staphylococcus aureus strains. The 3D crystal structure of SEB showed the location of the dominant epitopes. The fine-mapping of linear B-cell epitopes of the SEB antigen in this study will be useful to further understand anti-SEB immunity against MRSA infections and will be helpful to optimize MRSA vaccine designs that are based on the SEB antigen.

S. *aureus* is one of the most common causes of blood, skin and soft tissue infections worldwide [28], among which MRSA causes ventilator-assisted pneumonia, which induces significant morbidity and mortality [1]. SEB is a major virulence factor of S. *aureus* because of its direct role in toxic shock syndrome, which is induced by community-acquired methicillin-resistant Staphylococcus aureus (CA-MRSA) [9]. Although SEB has a conserved sequence and a stable structure, SEB is a potent toxin that acts as a lethal and incapacitating agent, which has limited native SEB as a vaccine candidate. Therefore, a nontoxic mutant of SEB may be a good candidate for MRSA vaccines.

Previous studies have confirmed that monoclonal antibodies of SEB can not only protect mice or human from common toxic shock syndrome that is induced by S. *aureus* infection [14]–[17] but also successfully treat other diverse diseases that are induced by S. aureus infections [21], all of which have demonstrated that the antibody response might play a major protective role in specific immunity against MRSA infection [20]. However, until now, no work has focused on the specific molecular basis and fine specificity of SEB antibodies, particularly their general characteristics, such as detailed epitope mapping of the protective antibody response to SEB. In the present study, we acquired a recombinant mutant SEB (rSEB), which was previously established [29]. The survival rate of immunized mice confirmed that rSEB was safe as a vaccine candidate. Then, we found that the rSEB vaccination could protect BALB/c mice from MRSA252 infection, with the assistance of an AlPO_4_ adjuvant. Because AlPO_4_ is a safe adjuvant in human vaccines, our research provides the possibility of rSEB as a vaccine candidate against human MRSA infection. High titer rSEB antisera were found in survival mice after MRSA infection, which further confirmed that the SEB humoral immune response played a key role against MRSA infection.

The identification of B-cell epitopes is rather important to immunotherapeutic and immunodetection applications because an epitope as the minimal immune unit is strong enough to elicit a potent humoral immune response without harmful side effects to the human body [30]. The most reliable methods for the identification of an epitope are monoclonal antibodies, X-ray crystallography and NMR techniques [26], [31]–[33]. To date, only five linear B cell epitopes of SEB have been identified by these methods, among which the truncated SEB mutant A was used to confirm SEB_252-261_ as an SEB epitope [25]; the anti-peptide monoclonal antibodies were used to reveal SEB_96-103_ as an SEB epitope [27]; the anti-TSST-1 monoclonal antibodies were used to reveal SEB_110-119_ as a cross epitope of SEB and TSST-1 [25]; and SEB_28-41_ and SEB_35-40_ were identified as SEB epitopes using the commercial anti-SEB monoclonal antibody ab53981 by a phage display approach [24]. However, these methods are time consuming, expensive for fine epitope mapping, and cannot identify immunodominant epitopes in the SEB antigen. Therefore, overlapping peptides, in combination with computational methods that are low cost and highly efficient, were rationally employed to identify linear B cell epitopes [34].

In this study, we mapped nearly all of the linear B-cell epitopes of SEB from MRSA252 using an ELISA with overlapping peptides. Our results suggested that SEB_97-114_, SEB_205-222_ and SEB_247-261_ induced dominant antibody responses. Among these three immunodominant peptides, we found that a novel B-cell epitope might exist in the region of SEB_205-222_, whereas two B-cell epitopes, SEB_252-261_ (INVEVHLTKK) and SEB_96-103_ (KNKNIDLF), had been previously reported in the regions of SEB_97–114_ and SEB_247-261_, respectively [25], [27]. Nevertheless, there were no detailed data regarding their immunogenicity or fine epitope mapping in previous studies. In our study, the antisera of three immunodominant peptides were prepared and provided strong responses to the native SEB antigen. Furthermore, these antisera were used to identify true epitopes in three peptides. We found that SEB_97-112_ had the higher response to antiserum of SEB_97–114_, which was notably stronger than the previously reported epitope, SEB_96-103_, and the response to the SEB_247-261_ antiserum corresponded with SEB_247-257_ rather than the previously reported SEB_252-261_. Importantly, a novel linear B-cell epitope, SEB_207-222_, first corresponded with SEB_205-222_. Moreover, all of these immunodominant epitopes were strongly recognized by sera from 36 MRSA-infected patients at the dilution of 1∶200 (data not shown).

As a superantigen, SEB can activate up to 20% of T-cells, which results in the induction of high levels of proinflammatory cytokines, including IL-2 and IFN-γ from T_H_1 cells [35]–[36] and TNF-α from activated APCs [37]. To evaluate the effect of the immunodominant epitope in vitro, all of the immunodominant epitope-specific antisera (anti-SEB_97-112_, anti-SEB_207-222_ and anti-SEB_247-257_) were acquired to determine the ability to inhibit SEB-induced T-cell proliferation and cytokine production from splenocytes of BALB/c mice.

For epitope-based vaccines, antigen conservation between different strains should be considered. To investigate the conservation of SEB_97–112_, SEB_207-222_, and SEB_247-257_ among different S. *aureus* strains, a sequence alignment of the corresponding regions on SEB was performed. The homology analysis indicated that SEB_97–112_ and SEB_207-222_ were well-conserved among different S. *aureus* strains, whereas SEB_247-257_ was not, which indicated that SEB_97–112_ and SEB_207-222_ might be good candidate molecules for vaccines that are based on SEB.

Furthermore, these three immunodominant epitopes were mapped on the 3D crystal structure of SEB. According to a previous study, the SEB molecule consists of two domains, one domain from 1–120 aa and the second domain from 127–239 aa [38]. As mentioned above, SEB_97–112_ was in the loop region of SEB within one domain, whereas SEB_207-222_ and SEB_247-257_ were in the β-slice region within the other domain. As described previously, within SEB_97–112_, N106, Y107, Y108 and Y109 are thought to participate in the binding of SEB and TCR [39]. Although no previous studies revealed the key amino acids in SEB activity within peptides SEB_207-222_ and SEB_247-257_, these two epitopes were on the surface of SEB, which indicated that these epitopes might induce antibody responses more easily than SEB_97–112_.

## Materials and Methods

### Ethics statement

All of the animal experiments were approved by the Animal Ethical and Experimental Committee of the Third Military Medical University (Chongqing; permit number 2011-04). All surgeries were performed under sodium pentobarbital anesthesia, and all efforts were made to minimize suffering.

### Animals and antigens

Six- to eight-week-old SPF female BALB/c mice were purchased from the Experimental Animal Center of Third Military Medical University (Chongqing,China). Recombinant mutant SEB (rSEB) was expressed in E. coli and purified as a C-terminal six-histidine-tagged (6×His) fusion protein, which was previously established [29]. Native SEB was purchased from the Academy of Military Science of China. The rSEB lost the ability to induce T cell mitogenesis and cytokine production in splenocytes of BALB/c mice (Figures S1–S4 in File S1).

### Peptide synthesis and KLH conjugation

Forty-two synthetic overlapping peptides, which spanned the entire length of the SEB of MRSA252, were constructed according to the reported sequence of SEB (Swiss-Prot ID: YP_185778.1). These peptides were separately synthesized, beginning with peptide no. 1 at the N-terminus and ending with peptide no. 42 at the C-terminus of SEB (ChinaPeptides Co., Ltd). The peptides consisted of 18 amino acid residues, with an overlap of twelve amino acids each. The purity of all of the above peptides was expected to be 90% or higher.

To locate the core sequences of the three major immunodominant epitopes of SEB, a series of synthetic truncated peptides were designed and synthesized by the same company. One or two amino acid residues were removed from either the N-terminal or the C-terminal of the three major immunodominant epitopes of SEB. For every immunodominant peptide of SEB, peptide KLH conjugations and core epitope KLH conjugations were performed by the same company. The negative control peptide, OVA_192-201_ (EDTQAMPFRV), was also synthesized by the same company.

The purity of all of the above peptides was expected to be 90% or higher.

### Immunization and MRSA challenge

Six- to eight-week-old SPF female BALB/c mice were purchased from the Experimental Animal Center of Third Military Medical University. Mice (n = 10) were immunized three times at two-week intervals with PBS alone, SEB alone or with SEB plus AlPO_4_ adjuvants (synthesized by Invitrogen), at 40 µg/mouse by intramuscular injection. To determine the survival rates after *S. aureus* infection, two weeks after the last immunization, BALB/c mice were anesthetized with sodium pentobarbital before injection and were intravenously injected with 0.84×10^9^ CFU of MRSA252. The survival rates were monitored for 14 days after infection. The conditions of the mice were monitored and recorded at 8, 16, and 24 hours every day. On the fifteenth day post-infection, mice were killed by CO_2_ asphyxiation. In the survival study, although the animals died as a direct result of the intervention, our research design included plans to consider humane euthanasia for mice that were observed to be suffering severe disease or that became moribund during the 14-day survival study.

To determine the immunogenicity of the three major immunodominant peptides, mouse anti-immunodominant peptide sera (anti-SEB_97-114_, anti-SEB_205-222_ and anti-SEB_247-261_) were produced in BALB/c mice by one intramuscular injection and two boost injections of 100 µg KLH-conjugated peptide or 100 µg rSEB plus 50 µL QuickAntibody adjuvant (Solarbio Company, China) at three-week intervals. Immune-serum was collected seven days after the final boost injection and was stored at −20°C until use.

To determine the immunogenicity of the immunodominant epitopes SEB_97-112_, SEB_207-222_ and SEB_247-257_, anti-immunodominant epitope sera (anti-SEB_97-114_, anti-SEB_205-222_ and anti-SEB_247-261_) were produced in BALB/c mice by one subcutaneous injection of 100 µg KLH-conjugated epitope in complete Freund's adjuvant (CFA) and two boost subcutaneous injections of 100 µg KLH-conjugated epitope in incomplete Freund's adjuvant (IFA) at two-week intervals. Immune-serum was collected seven days after the final boost injection and was stored at −20°C until use.

All animals in the survival study were sacrificed by CO_2_ asphyxiation when they became moribund, as defined by a combination of ruffled fur, hunched back and dulled response to stimuli, such as finger probing, when we monitored and recorded the condition of the mice at 8, 16, and 24 hours every day. At the completion of all experiments, survivors were sacrificed by CO_2_ overdose in accordance with IACUC policy.

### ELISA to detect anti-SEB antibodies and anti-immunodominant epitope antibodies

To determine the anti-SEB titers in immunized animal serum samples, microtiter plates were coated with 1 µg/well of native SEB (China Academy of Military Medical Science) in 100 µL of carbonate buffer overnight at 4°C. Wells were then blocked with 5% skim milk in PBS. After the wells were washed, serial dilutions of sera were incubated in the wells for 1 h at room temperature. After any unbound material was washed off, a peroxidase-conjugated goat anti-mouse IgG (Southern Biotech, Birmingham, AL) was added for 1 h. After being washed, plates were incubated with a TMB substrate (BioFX) at room temperature. The enzymatic reactions were stopped by the addition of 2 M sulfuric acid, and endpoint absorbances were read at 450 nm. Titers were defined as the last serum dilution with an absorbance that was double that of sera from animals that received PBS only.

All immunizations that induced antibodies against the KLH-conjugated peptide or against the KLH-conjugated epitope were measured identically by an enzyme-linked immune-sorbent assay (ELISA) of sera that were collected seven days after the final boost immunization.

### Epitope mapping with synthetic overlapping peptides

The peptides were dissolved in dimethyl sulfoxide (DMSO) at 0.5 mg/mL and diluted in hydrogen bicarbonate buffer (pH 9.6) to 5 µM. Serum samples from SEB-immunized mice were diluted 1∶300. Serum samples from peptide-KLH-immunized mice were diluted at different titers (as shown in Figure 3). Non-specific binding was prevented by blocking the coated microtiter plates with phosphate buffered saline (PBS, pH 7.4), which contained 5% skim milk, for 1 h. As secondary antibodies, peroxidase-conjugated goat anti-mouse IgG antibodies (Dianova, Hamburg, Germany) were used at a dilution of 1∶3000. Optimal peptide concentrations and serum dilutions were determined using serial dilutions. The results of the ELISA were given as absorbance values. The normal values for each peptide were calculated by testing sera from normal mice. The values that were above the mean absorbance value of these sera plus three times the standard deviation were defined as positive.

### *In Vitro* SEB Neutralization

Splenic mononuclear cells from BALB/c were isolated using Ficoll-Paque Plus (GE Healthcare, Uppsala, Sweden) gradient centrifugation, washed 2× with PBS and resuspended in 90% RPMI 1640, 10% fetal bovine serum (FBS), 55 µM 2-ME (all from Invitrogen, Carlsbad, CA), and 20 mg/ml gentamicin (Sigma-Aldrich, St. Louis, MO). In total, 50 µl of the indicated concentrations of SEB and 50 µl of the indicated concentrations of anti-immunodominant epitope antibodies/or the indicated concentrations of normal sera were incubated in the wells of 96-well flat-bottom plates (at 37°C in 7.5% CO_2_) for 1 hours and then cell suspensions (1×10^6^ cells/well) were added. Each condition was run in 2× triplicate with two equal parts. In one part, the supernatants of cell cultures that were collected at 48 hrs after the indicated treatment were tested using a Bio-plex cytokine ELISA (Dakewe Biotech Co., Ltd.) according to the manufacturer's instructions to determine the levels of IL-2, IFN-γ and TNF-α. In the other part, the treated cells were incubated (at 37°C in 7.5% CO_2_) for 48 hours and then pulsed with 1 µCi ^3^H-thymidine (Amersham/GE Healthcare) per well, incubated an additional 18 hours and then harvested. The incorporated radioactivity was measured using liquid scintillation counting, and stimulation indices (SI) were calculated as follows: SI = (Average net CPM from SEB treated cultures)/(Average net CPM from untreated cultures).

### Sequence alignment and localization of immunodominant peptides on SEB

SEB sequences from different S. *aureus* strains were retrieved from the GenBank database for alignment using the MEGA software. The 3D structure of SEB was previously reported [12]. Immunodominant peptides were mapped against the 3D structure of SEB (PubMed protein database) using the PyMOL 1.1 program with the crystal structure of SEB.

### Statistical analyses

All statistical analyses were performed using an unpaired Student's t-test in the software SPSS 13.0. Graphs were created using the Prism software (GraphPad Prism 5, San Diego, CA). The data were presented as the means plus standard deviations from three independent experiments. P values<0.05 were considered significant.

## Supporting Information

File S1 Splenic mononuclear cells from BALB/c mice were cultured in 96-well flat-bottom tissue culture plates and incubated at 37°C in 5% CO_2_. Various concentrations (50 ng/mL, 100 ng/mL and 200 ng/mL) of SEB or rSEB were added. Cells in medium alone without treatment were used as controls. Each condition was run in 2× in triplicate with two equal parts. In one part, the supernatant of cell cultures collected at 48 hrs after the indicated treatment were tested using a Bio-plex cytokine ELISA (Dakewe Biotech Co., Ltd.) according to the manufacturer's instructions to determine the levels of IL-2, IFN-γ and TNF-α. In the other part, the treated cells were incubated (at 37°C in 7.5% CO_2_) for 48 hours, then pulsed with 1 µCi ^3^H-thymidine (Amersham/GE Healthcare) per well, incubated an additional 18 hours and then harvested. The incorporated radioactivity was measured using liquid scintillation counting, and stimulation indices (SI) were calculated. **Figure S1.** Various concentrations of SEB could induce T cell mitogenesis, whereas rSEB lost this ability **Figure S2.** Various concentrations of SEB could induce IL-2 production from splenic lymphocytes of BALB/c mice, whereas rSEB lost this ability. **Figure S3**. Various concentrations of SEB could induce IFN-γ production from splenic lymphocytes of BALB/c mice, whereas rSEB lost this ability. **Figure S4.** Various concentrations of SEB could induce TNF-α production from splenic lymphocytes of BALB/c mice, whereas rSEB lost this ability.
(TIF)

## References

[1] Otto M (2012) MRSA virulence and spread(2012). *Cellular* Microbiology 14: 1513–1521. 22747834 10.1111/j.1462-5822.2012.01832.x PMC3443268

[2] Demei Z, Fu W, Fupin H, Xiaofei J, Yuxing N, et al (2011) China's CHINET surveillance of bacterial resistance In 2010. Chinese Journal of infection and chemotherapy 5: 326–329.

[3] Nakamura A, Miyake K, Misawa S, KunoY, Horii T, et al (2012) Association between antimicrobial consumption and clinical isolates of methicillin-resistant Staphylococcus aureus: a 14-year study. J Infect Chemother 18: 90–95. 21947660 10.1007/s10156-011-0302-6

[4] Klevens RM, Morrison MA, Nadle J, Petit S, Gershman K, et al (2007) Invasive methicillin-resistant Staphylococcus aureus infections in the United States. JAMA 298: 1763–1771. 17940231 10.1001/jama.298.15.1763

[5] Daum RS, Spellberg B (2012) Progress Toward a Staphylococcus aureus Vaccine. Vaccines 54: 560–567. 10.1093/cid/cir828 PMC3404717 22186773

[6] Irina VP, Ellen JB, Victor ER (2010) Staphylococcal Enterotoxins. Toxins 2 2177–2197. 22069679 10.3390/toxins2082177 PMC3153290

[7] Fluer FS (2012) Staphylococcus enterotoxins, their properties and role as pathogenicity factors. Zh Mikrobiol Epidemiol Immunobiol 2: 99–108. 22693820

[8] Dinges MM, Orwin PM, Schlievert PM (2000) Exotoxins of Staphylococcus aureus. Clin. Microbiol. Rev 13: 16–34. 10627489 10.1128/cmr.13.1.16-34.2000 PMC88931

[9] Kashiwada T, Kikuchi K, Abe S, Kato H, Hayashi H, et al (2012) Staphylococcal Enterotoxin B Toxic Shock Syndrome Induced by Community-acquired Methicillin-resistant Staphylococcus aureus (CA-MRSA). Intern Med.51: 3085–3088. 23124156 10.2169/internalmedicine.51.7295

[10] DeVries AS, Lesher L, Schlievert PM, Rogers T, Villaume LG, et al (2011) Staphylococcal toxic shock syndrome 2000–2006: epidemiology, clinical features, and molecular characteristics. PLoS One.6: e22997. 21860665 10.1371/journal.pone.0022997 PMC3157910

[11] Breuer K, Wittmann M, Bosche B, Kapp A, Werfel T (2000) Severe atopic dermatitis is associated with sensitization to staphylococcal enterotoxin B (SEB). Allergy 55: 551–555. 10858986 10.1034/j.1398-9995.2000.00432.x

[12] Rossi RE, Monasterolo G (2004) Prevalence of serum IgE antibodies to the Staphylococcus aureus enterotoxins (SAE, SEB, SEC, SED, TSST-1) in patients with persistent allergic rhinitis. Int Arch Allergy Immunol 133: 261–266. 14976395 10.1159/000076833

[13] Karauzum H, Chen G, Abndou L, Mahmoudieh M, Boroun AR, et al (2012) Synthetic human monoclonal antibodies towards staphylococcal enterotoxin B (SEB) protective against toxic shock syndrome. J. Biol. Chem 287: 25203–25215.13. 22645125 10.1074/jbc.M112.364075 PMC3408135

[14] Larkin EA, Stiles BG, Ulrich RG (2010) Inhibition of toxic shock by human monoclonal antibodies against staphylococcal enterotoxin B. PLoS One 10: 1–9. 10.1371/journal.pone.0013253 PMC2952590 20949003

[15] Tilahun ME, Kwan A, Natarajan K, Quinn M, Tilahun AY, et al (2011) Chimeric anti-staphylococcal enterotoxin B antibodies and lovastatin act synergistically to provide in vivo protection against lethal doses of SEB. PLoS One 11 1–8: 16. 10.1371/journal.pone.0027203 PMC3216929 22102880

[16] Varshney AK, Wang X, Cook E, Dutta K, Scharff MD, et al (2011) Generation, characterization, and epitope mapping of neutralizing and protective monoclonal antibodies against staphylococcal enterotoxin B-induced lethal shock. J. Biol. Chem 286: 9737–9747. 21233204 10.1074/jbc.M110.212407 PMC3059042

[17] Karauzum H, Chen G, Abndou L, Mahmoudieh M, Boroun AR, et al (2012) Synthetic Human Monoclonal Antibodies toward Staphylococcal Enterotoxin B(SEB) Protective against Toxic Shock Syndrome. The Journal of biological chemistry 30: 25203–25215. 10.1074/jbc.M112.364075 PMC3408135 22645125

[18] Balali-Mood M, Moshiri M, Etemad L (2013) Medical aspects of bio-terrorism. Toxicon.69: 131–142. 23339855 10.1016/j.toxicon.2013.01.005

[19] Rusnak JM, Kortepeter M, Ulrich R, Poli M, Boudreau E (2004) Laboratory exposures to staphylococcal enterotoxin B. Emerg. Infect Dis.10: 1544–1549. 10.3201/eid1009.040250 PMC3320288 15498154

[20] Birch H (2011) Antibodies could lead to MRSA vaccine (2011) Chemistry World. 8: 21–21.

[21] Varshney AK, Wang X, Scharff MD, MacIntyre J, Zollner RS, et al (2013) Staphylococcal Enterotoxin B–Specific Monoclonal Antibody 20B1 Successfully Treats Diverse Staphylococcus aureus Infections. The Journal of Infectious Diseases 208 2058–66. 23922375 10.1093/infdis/jit421 PMC3836467

[22] Lindsay CD, Griffiths GD (2013) Addressing bioterrorism concerns: options for investigating the mechanism of action of Staphylococcus aureus enterotoxin B. Hum Exp Toxicol 32: 606–619. 23023027 10.1177/0960327112458941

[23] Krakauer T (2010) Therapeutic Down-Modulators of Staphylococcal Superantigen Induced Inflammation and Toxic Shock. Toxins 2: 1963–1983. 22069668 10.3390/toxins2081963 PMC3153276

[24] Urushibata Y, Itoh K, Ohshima M, Seto Y (2010) Generation of Fab fragment-like molecular recognition proteins against staphylococcal enterotoxin B by phage display technology. Clin Vaccine Immunol 17: 1708–1. 20844088 10.1128/CVI.00229-10 PMC2976098

[25] Nishi JI, Kanekura S, Takei S, Kitajima I, Nakajima T, et al (1997) B Cell Epitope Mapping of the Bacterial Superantigen Staphylococcal Enterotoxin B. The Journal of Immunology 158: 247–254. 8977196

[26] Bhatti AR, Micusanb VV (1999) Production and characterization of anti-peptide monoclonal antibodies with specificity for staphylocokal enterotoxins A and B. Journal of Microbiological Methods 35: 143–149. 10192046 10.1016/S0167-7012(98)00110-9 PMC7133826

[27] Kum WWS, Chow AW (2001) Inhibition of Staphylococcal Enterotoxin A–Induced Superantigenic and Lethal Activities by a Monoclonal Antibody to Toxic Shock Syndrome Toxin–1. The Journal of Infectious Diseases 183: 1739–48. 11372026 10.1086/320732

[28] Landrum ML, Neumann C, Cook C, Chukwuma U, Ellis MW, et al (2012) Epidemiology of Staphylococcus aureus Blood and Skin and Soft Tissue Infections in the US Military Health System, 2005-2010. JAMA 308: 50–59. 22760291 10.1001/jama.2012.7139

[29] Inskeep TK, Stahl C, Odle J, Oakes J, Hudson L, et al (2010) Oral Vaccine Formulations Stimulate Mucosal and Systemic Antibody Responses against Staphylococcal Enterotoxin B in a Piglet Model. Clinical and Vaccine Immunology 17: 1163–1169. 20554806 10.1128/CVI.00078-10 PMC2916245

[30] Varshney AK, Wang X, Cook E, Dutta K, Scharff MD, et al (2011) Generation, characterization, and epitope mapping of neutralizing and protective monoclonal antibodies against staphylococcal enterotoxin B-induced lethal shock. J Biol Chem 286: 9737–9747. 21233204 10.1074/jbc.M110.212407 PMC3059042

[31] Irving MB, Pan O, Scott JK (2001) Random-peptide libraries and antigen-fragment libraries for epitope mapping and the development of vaccines and diagnostics. Current Opinion in Chemical Biology 5: 314–324. 11479124 10.1016/S1367-5931(00)00208-8 PMC7129944

[32] Rux JJ, Burnett RM (2000) Type-specific epitope locations revealed by X-ray crystallographic study of type 5 hexon. Molecular Therapy 1: 18–30. 10933908 10.1006/mthe.1999.0001

[33] Mayer M, Meyer B (2001) Group epitope mapping by saturation transfer difference NMR to identify se adenovirus gments of a ligand in direct contact with a protein receptor”. Journal of the American Chemical Society 123: 6108–6117. 11414845 10.1021/ja0100120

[34] Komatsu N, Jackson HM, Chan KF, Oveissi S, Cebon J, et al (2013) Fine-mapping naturally occurring NY-ESO-1 antibody epitopes in melanoma patients' sera using short overlapping peptides and full-length recombinant protein. Molecular Immunology 54: 465–471. 23454162 10.1016/j.molimm.2013.01.014

[35] Arad G, Hillman D, Levy R, Kaempfer R (2001) Superantigen antagonist blocks Th1 cytokine gene induction and lethal shock. J Leukoc Biol 69: 921–927. 11404377

[36] Kappler J, Kotzin B, Herron L, Gelfand E, Bigler R, et al (1989) V beta-specific stimulation of human T cells by staphylococcal toxins. Science 244: 811–813. 2524876 10.1126/science.2524876

[37] Baum D, Yaron R, Yellin MJ (1998) TNF-alpha, not CD154 (CD40L), plays a major role in SEB-dependent, CD4(+) T cell-induced endothelial cell activation in vitro. Cell Immunol 190: 12–22. 9826442 10.1006/cimm.1998.1380

[38] Kappler JW, Herman A, Clements J, Marrack P (1992) Mutations defining functional regions of the superantigen staphylococcal enterotoxin B. J Exp Med 175: 387–396. 1370682 10.1084/jem.175.2.387 PMC2119125

[39] Hurley JM, Shimonkevitz R, Hanagan A, Enney K, Boen E, et al (1995) Identification of class II major histocompatibility complex and T cell receptor binding sites in the superantigen toxic shock syndrome toxin1. J Exp Med 181: 2229–2235. 7760008 10.1084/jem.181.6.2229 PMC2192066

