# Supplementary figures and images for: Fine-Mapping of Immunodominant Linear B-Cell Epitopes of the Staphylococcus Aureus SEB Antigen Using Short Overlapping Peptides
Source: PLoS One. 2014 Mar 5;9(3):e90445. doi: 10.1371/journal.pone.0090445 (PMC3943954; doi:10.1371/journal.pone.0090445)

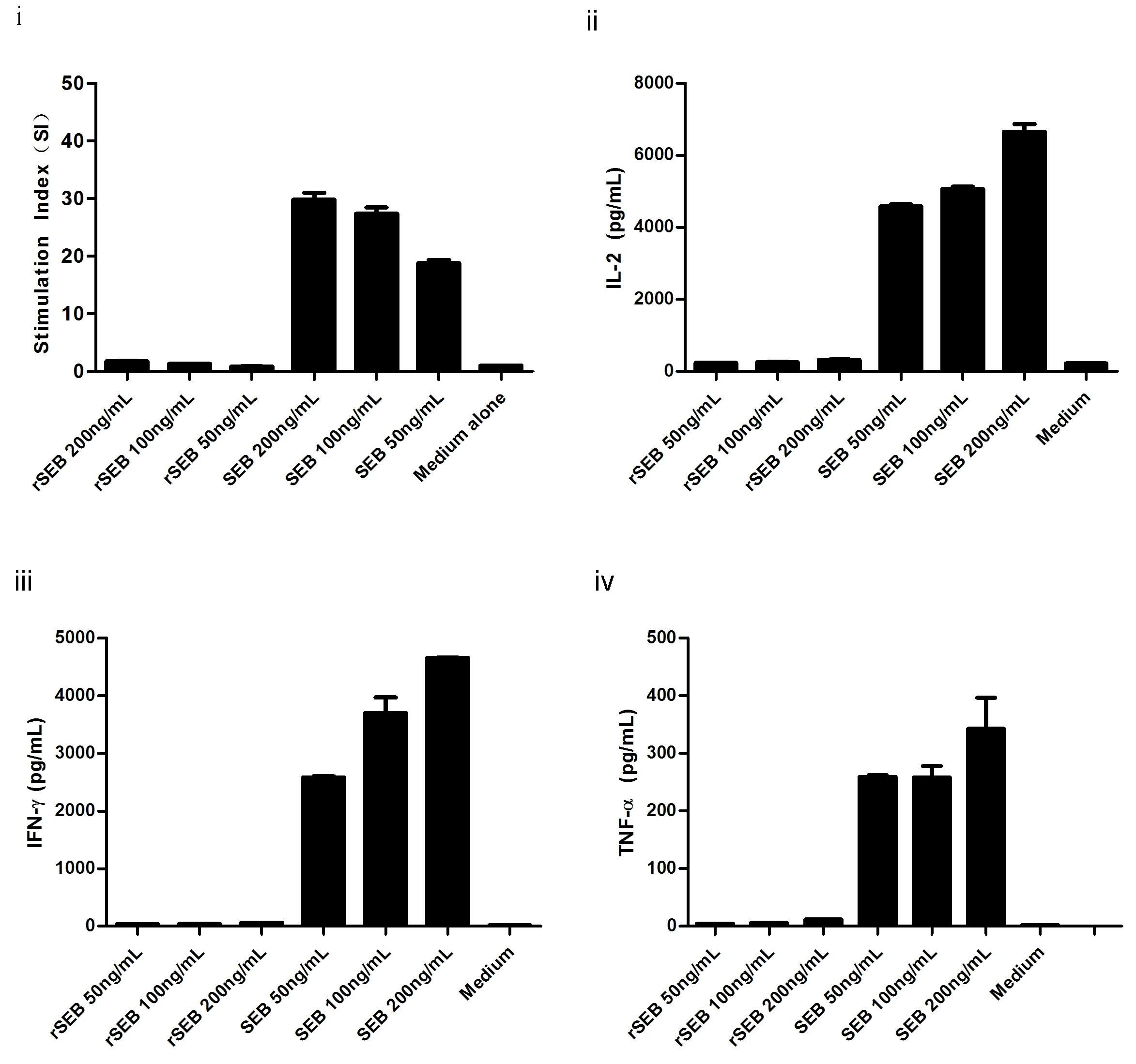

Supplement: File S1 — Splenic mononuclear cells from BALB/c mice were cultured in 96-well flat-bottom tissue culture plates and incubated at 37°C in 5% CO2. Various concentrations (50 ng/mL, 100 ng/mL and 200 ng/mL) of SEB or rSEB were added. Cells in medium alone without treatment were used as controls. Each condition was run in 2× in triplicate with two equal parts. In one part, the supernatant of cell cultures collected at 48 hrs after the indicated treatment were tested using a Bio-plex cytokine ELISA (Dakewe Biotech Co., Ltd.) according to the manufacturer's instructions to determine the levels of IL-2, IFN-γ and TNF-α. In the other part, the treated cells were incubated (at 37°C in 7.5% CO2) for 48 hours, then pulsed with 1 µCi 3H-thymidine (Amersham/GE Healthcare) per well, incubated an additional 18 hours and then harvested. The incorporated radioactivity was measured using liquid scintillation counting, and stimulation indices (SI) were calculated. Figure S1. Various concentrations of SEB could induce T cell mitogenesis, whereas rSEB lost this ability Figure S2. Various concentrations of SEB could induce IL-2 production from splenic lymphocytes of BALB/c mice, whereas rSEB lost this ability. Figure S3. Various concentrations of SEB could induce IFN-γ production from splenic lymphocytes of BALB/c mice, whereas rSEB lost this ability. Figure S4. Various concentrations of SEB could induce TNF-α production from splenic lymphocytes of BALB/c mice, whereas rSEB lost this ability. (TIF) [file pone.0090445.s001.tif]
